# Ultrasonic pretreatment and drying temperature-induced modifications of three pectin fractions affect the microstructure and textural properties of dried grapes

**DOI:** 10.1016/j.fochx.2025.102633

**Published:** 2025-06-04

**Authors:** Yadong Xiao, Yiwen Yang, Yayuan Xu, Lei Feng, Meimei Nie, Liying Niu, Chunquan Liu, Chunju Liu, Dajing Li, Zhifang Yu

**Affiliations:** a College of Food Science and Technology, Nanjing Agricultural University, Nanjing 210095, China; b Institute of Agro-product Processing, Jiangsu Academy of Agricultural Sciences, Nanjing 210014, China; c College of Life Sciences, Northeast Forestry University, Harbin 150040, China

**Keywords:** Grapes, Pretreatment, Vacuum drying, Texture properties, Pectin, PCA

## Abstract

The effect of ultrasonic pretreatment on grapes' microstructure, texture, characteristics of three pectin fractions were investigated over vacuum drying temperature ranging from 65 to 85 °C. Ultrasonic treated samples exhibited a better microstructure and texture quality than with vacuum drying alone. High drying temperature led to increased water-soluble pectin (WSP, from 23.85 to 25.66 mg/g AIR) and chelate-soluble pectin (CSP, from 1.92 to 2.87 mg/g AIR) content, decreased sodium carbonate-soluble pectin (NSP, from 12.11 to 4.43 mg/g AIR) content. Within pectin fractions, monosaccharide content and molecular weight both decreased as the drying temperature increased after ultrasonic pretreatment. Characterization by Fourier transform infrared spectroscopy, X-ray diffraction, circular dichroism, and scanning electron microscopy revealed differences between pectin fractions in terms of structures. Principal component analysis and correlation analysis indicated that the changes in galacturonic acid content and sugar ratio of WSP, NSP fractions influenced the texture properties of dried grapes.

## Highlights

• Ultrasonic pretreatment samples showed a more porous microstructure and crisper texture.
• Ultrasonic pretreatment and high temperature led to the modifications of three pectin fractions.
• Differences in microstructure of three pectin fractions were observed.
• The texture properties of dried grapes were strongly correlated with WSP and NSP.
• An innovative drying method of crispy grapes was established.

## 1 Introduction

Grapes are delicious and have high nutritive value due to containing abundant phenols, sugars, organic acids, and other such compounds (Ramchandani, Chettiyar, & Pakhale, 2010). However, after harvesting, grapes easily lose water, soften, and decay, which is not conducive to storage and transportation. Drying is currently one of the most widely used techniques for processing grapes on account of its simplicity, low equipment requirements, and the ease of long-term transportation and storage of dried products. Moreover, in recent years, crispy chips/grains prepared from fruits have been gaining more and more consumer attention due to their nutritional content and the pleasant crispy mouthfeel they provide.

Texture is essential in influencing the overall quality and consumer acceptance of dried fruit products. During the drying process, the internal structure and compounds change due to the moisture inside the fruits continuously diffusing out, and the internal structure of dried products generally becomes dense. The specific textural properties of dried fruits are substantially related to their composition and the structural properties of the chemical substances they contain (Feng et al., 2022; Liu et al., 2021; Prosapio & Norton, 2018), especially cell wall polysaccharides. Pectin is the most important cell wall polysaccharide and is mainly present in the middle lamella of the cell wall (Zdunek, Pieczywek, & Cybulska, 2021), where it plays a crucial role in promoting cell adhesion and strength in plant tissues. Notably, the unique chain structure of pectin makes it extremely susceptible to external factors such as temperature and pressure during processing (Bordoloi, Kaur, & Singh, 2012). Therefore, it is an interesting work to understand how pectin changes affect the textural properties of dried fruit products.

The pectin in fruit mainly occurs in three bonding states: water-soluble pectin (WSP), trans-cyclohexane 1,2-diaminetetraacetic acid (CDTA)-soluble pectin (CSP), and sodium carbonate-soluble pectin (NSP) (Oshima, Kato, & Imaizumi, 2021; Yu, Li, Hu, Wang, & Bi, 2023). WSP is loosely bound to other cell-wall polysaccharides through non-covalent or non-ionic bonds, CSP is cross-linked to the cell wall through interaction with ions such as calcium and dilute acids, and NSP is covalently bound to the cell wall (Pan et al., 2022). Degradation of pectin destroys the three-dimensional cell network structure, reduces the strength of cross-linking between adjacent cells, and changes the texture quality of plant-based products (Sila et al., 2009). Recently, the application of ultrasonic pretreatment to enhance drying efficiency and products quality has garnered considerable attention (Waghmare et al., 2022). And ultrasonic pretreatment has been shown to modify the cell wall polysaccharides structure in several fruits and vegetables (Guo, Liu, Li, Liu, et al., 2022; Luo et al., 2024; Zhang et al., 2024). An increase in WSP and a decrease in CSP and NSP concentrations were found in mulberry samples with ultrasonic pretreatment, and the Mw of all three pectin fractions declined over the course of extended ultrasonic exposure (Wang et al., 2024). The same phenomenon was observed in strawberry pectin (Xing et al., 2023) and citrus pectin (Qiu, Cai, Wang, & Yan, 2019). Meantime, our previous study also confirmed ultrasonic pretreatment to influence the content and structure of three pectin fractions in fresh grapes (Yang et al., 2023). Therefore, the effects of ultrasonic pretreatment on pectin characteristics of dried grapes requires further investigation to achieve a understanding of final products quality.

The changes in pectin characteristics are closely related to the texture properties of dried fruit products (Yu et al., 2023). In apricots, high-humidity hot air impingement blanching likewise leads to pectin transformation and degradation, causing softening of the dried product (Deng et al., 2018). Ji et al. (2024) also confirmed that CSP and NSP content is positively correlated with the hardness and crispness of dehydrated jujube products. As a common drying technique, vacuum drying has the benefits of lower drying temperature, lower oxygen content, and fast drying velocity compared to other drying methods, and thus can better maintain the nutrient quality, color, and shape of dried products. Studies have shown that vacuum drying enhances color retention and crispness in kiwi fruit slices and dried blueberries. However, few studies have investigated the relationship between cell wall polysaccharide characteristics and the microstructure, textural quality of vacuum-dried grapes.

Hence, the purpose of this study was to investigate: (i) the effects of vacuum drying(with or without ultrasonic pretreatment) on the microstructure and textural properties of dried grapes; (ii) the galacturonic acid (GalA) content, monosaccharide composition and structural characteristics of three pectin fractions in dried grapes; and (iii) the relationship between pectin composition and textural properties through correlation analysis and principal component analysis (PCA).

## 2 Materials and methods

### 2.1 Materials and chemicals

Grapes (*Vitis vinifera*, ‘Summer Black’) harvested in July and August were purchased from a local market (Xiaolingwei, Xuanwu District, Nanjing, China). Grapes were stored in a refrigerator at 4 °C until the experiment. High-performance liquid chromatography (HPLC) grade methanol, acetonitrile, trifluoroacetic acid, and 1-phenyl-3-methyl-5-pyrazolinone were obtained from TEDIA Reagent Company (Ohia, USA).

Monosaccharide standards (L-rhamnose, D-galactose at purity of 99 %) were purchased from Sigma-Aldrich Co., Ltd. (St, Louis, Mo, USA). L-arabinose, d-glucose, D-glucuronic acid and D-mannose (HPLC ≥98 %) were purchased from Shanghai yuanye Bio-Technology Co., Ltd. (Shanghai, China). Dialysis tubing (MD77MM, 3.5 KDa) was obtained from Viskase Companies, Inc. (Lombard Illinois, USA). Other chemicals and reagents used in this study were analytical grade.

### 2.2 Sample preparation

**Fig. 1:**
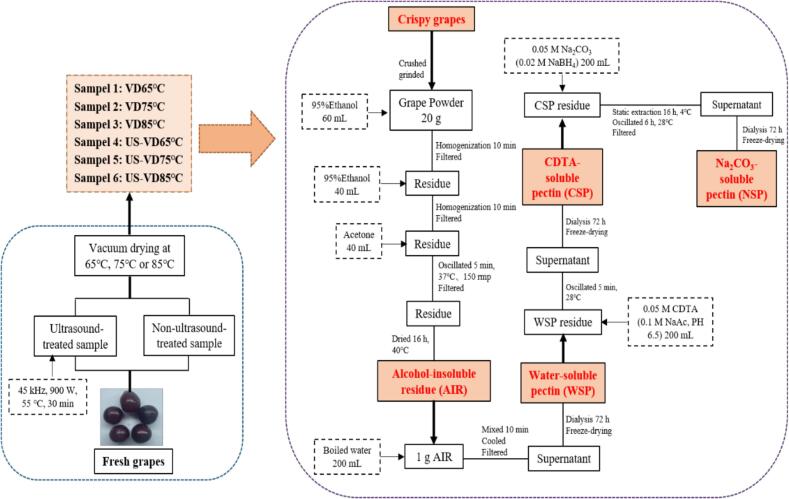
Sample preparation process and three pectin fraction extraction process.

The fresh grapes were destemmed and cleaned, and 500 g of grapes allotted per sample. The initial moisture content of the sample was estimated according to the method of AOAC (2002) and found to be 89.20 % ± 0.21 % (wet basis, w. b.). We prepared ultrasonic-treated and untreated samples. A 900 W ultrasonic bath (Kun Shan Ultrasonic Instruments Co., Ltd., Suzhou, China) was used for ultrasonic-treated samples. The grapes were immersed in 2000 mL distilled water (the sample to water ratio was 1:4, m/*v*), then the treatment was carried out at 45 kHz frequency maintaining the temperature at 55 °C for 30 min (Yang et al., 2023). After that, grapes with or without ultrasonic pretreatment were dried inside a vacuum drying oven (STIK CO., LTD, IVOS-60, Shanghai, China) with a temperature of 65 °C, 75 °C, and 85 °C at a vacuum pressure of not less than 0.08 MPa. The moisture content of the final dried grapes was less than 8 % (w. b.). All experiments were performed in triplicate. Samples are labeled according to treatment as follows: VD65, US-VD65, VD75, US-VD75, VD85, US-VD85, as shown in Fig. 1.

### 2.3 Microstructural analysis of dried grapes

#### 2.3.1 Determination of cellular morphology

Cell morphology analysis of dried grapes was conducted using a Nikon Eclipse E 200 light microscope (Nikon Corporation, Tokyo, Japan). Referring to the method of Liu et al. (2021) with minor modification, the samples were cut into cubes no larger than 0.6 cm per side and then fixed in formalin-acetic acid-alcohol solution (50 % ethanol, 5 % formaldehyde [37 % ∼ 40 %], and 5 % glacial acetic acid, *v*/v/v) for more than 24 h. After that, the cubes were dehydrated, waxed, embedded, sliced to a thickness of 20 μm, stained with periodic Acid-Schiff, and finally observed using a light microscope at 25 °C.

#### 2.3.2 Determination of microstructure

The microstructure of dried grapes was examined with a scanning electron microscope (EVO-LS10, ZESS Company, Jena, Germany) according to the method described by Guo et al. (2022). Hand-cut cross-sectional samples were attached to a sample stage coated with conductive adhesive and sputtered immediately with a layer of gold under vacuum condition. Observation was performed using an accelerating voltage of 10 kV and the images were recorded at a magnification of 50 × .

#### 2.3.3 Determination of cell area, cell diameter, number of cells, fractal dimension, and porosity

Based on the optical microscope images obtained in 2.3.1, the cell area, cell diameter, number of cells, and fractal dimension of dried grapes were determined according to the method described by Yuan et al. (2023) with some modification. The image processing process is shown in S-Fig. 1. After grayscale conversion, the image was converted into a binary image. The cell structure parameters were calculated using the Image-Pro Plus 6.0 software (Media Cybernetics, USA). And the porosity was determined by first flagging cell pores and walls with black and white respectively, then calculating the proportion of pores in the image.

### 2.4 Texture analysis

The textural properties of crispy grapes were evaluated in terms of four characteristic indicators, namely hardness, rupture distance, fracture slope, and rupture energy. These indicators were obtained by a CT3 25 K texture analyzer (Brookfield Ltd., MA, USA) with probe model TA 44 and in the compression testing mode. Hardness is defined as the maximum force during the compression rupture, which is indicated by the peak value of the force-time curve. Rupture distance is the distance the probe moves when the first rupture emerges. The fracture slope is the ratio of the first rupture force to the corresponding time (time on the corresponding abscissa). Finally, the rupture energy is the energy consumed in the probe during one compression, which is defined as the area enclosed by the curve and the abscissa. Twenty grape particles were measured for each drying condition, and the test parameters consisted of a pressing distance 50 % of the initial sample height and a testing speed of 1 mm/s. After obtaining the four indicators, the texture characteristics index (Te) was calculated using the following equation (Mu, Jin, Zheng, & Liu, 2010):

$$\mathrm{Te}=\frac{\sqrt{K^{2}-S^{2}-W^{2}}}{H}$$

where Te denotes the texture characteristics index, *H* the hardness (g), *S* the rupture distance (mm), *K* the fracture slope (g/s), and *W* the rupture energy (g·s).

### 2.5 Preparation of pectin

#### 2.5.1 Preparation of alcohol insoluble residue (AIR)

The AIR was prepared from dried grapes according to the method of Mcfeeters and Armstrong (1984) with some modifications. Briefly, each dried grape was crushed and ground into powder, of which 20 g was dissolved in 60 mL 95 % ethanol and homogenized for ten minutes. After filtration, the residue was dissolved in 40 mL 95 % ethanol and homogenized for another ten minutes. Next, the residue was dissolved in 40 mL acetone and oscillated in an incubator for five minutes at 37 °C and 150 rpm, after which filtering was again performed. The resulting residue was then dried at 40 °C for 16 h, from which the AIR was obtained. Finally, the AIR was placed in a dryer for subsequent extraction.

#### 2.5.2 Extraction of three pectin fractions

The three pectin fractions were separated from the AIR according to the method of Sila, Doungla, Smout, Loey, and Hendrickx (2006) with some modifications. WSP fraction was prepared by dissolving 1.000 g AIR in 200 mL boiled water, mixing for ten minutes, then cooling and filtering the solution. The supernatant was then dialyzed for 72 h, and finally freeze-dried to obtain WSP. The CSP fraction was prepared by dissolving the residue from WSP preparation in 200 mL 0.05 M CDTA solution (with 0.1 M sodium acetate, pH = 6.5), oscillating it for 15 min at 28 °C in an oscillator, then filtering the solution. This supernatant was also dialyzed for 72 h, then lyophilized to obtain CSP. The NSP fraction was prepared by dissolving the residue from CSP preparation in 200 mL 0.05 M sodium carbonate solution (with 0.02 M sodium borohydride) and letting it stand for 16 h at 4 °C. Afterward, the solution was shocked for six hours at 28 °C in a water bath oscillator, then filtered. Finally, the supernatant was dialyzed for 72 h, and then lyophilized to obtain NSP. A summary of this preparation process is illustrated in Fig. 1.

### 2.6 Structural characteristics analysis of three pectin fractions in dried grapes

#### 2.6.1 Determination of galacturonic acid (GalA)

The GalA content in three pectin fractions was determined by the carbazole‑sulfuric acid method of Galambos (1967) and Blumenkrantz and Asboe-Hansen (1973). Briefly, 1.5 mL of sample solution containing pectin was prepared in a glass tube with a lid, and 1.5 mL of distilled water and 0.75 mL of carbazole solution (10 mL methanol dissolved in 50 mg carbazole) were added sequentially into it. Next, 15 mL of sulfuric acid was added quickly into the tube under continuous shaking, after which the lid was sealed and the solution was shaken well. The mixture was then heated for 20 min in boiling water (80 °C). After cooling to room temperature, the absorbance of the solution was measured at 540 nm by an ultra*v*iolet and visible spectrophotometer (UV-6300, Shanghai, China). The standard curve obtained in this experiment was y = 0.0059× + 0.0494 (R^2^ = 0.9940).

#### 2.6.2 Determination of monosaccharide compositions

The monosaccharide composition and content of each grape pectin fraction was determined using the 1-phenyl-3-methyl-5-pyrazolone (PMP) pre-column derivatization method described by Dai, Wu, Chen, et al. (2010). Notably, the derivatization of all hydrolysate and standard monosaccharide samples must be carried out simultaneously under the same conditions.

The Aglient 1200 system (Aglient Technologies, California, USA) was used for monosaccharide analysis. Separation of six monosaccharides was performed on an RP-C18 column (4.6 × 250 mm, 5 μm Venusil, USA) using a mobile phase of 0.1 M phosphate buffer solution (PBS) (pH = 6.7)/acetonitrile (83/17, *v*/v) with a flow rate of 1.0 mL/min. The column temperature was 30 °C, and the detection wavelength was 245 nm.

A range of concentrations (0.01–5 mg/L) of each monosaccharide (rhamnose, Rha; arabinose, Ara; galactose, Gal; glucose, Glu; glucuronic acid, GluA; mannose, Man) were used as a standard curve for quantification (S-Table 1).

**Table 1:** Effects of different drying conditions on the Cell Area, Cell Diameter, Number of Cells, and Fractal Dimension of dried grapes.

Drying method	Drying temperature	Cell area (μm^2^)					Cell diameter (μm)					Numbers of cells	Fractal dimension
		Minimum value	Maximum value	Average value	Standard deviation	Coefficient of variation (%)	Minimum value	Maximum value	Average value	Standard deviation	Coefficient of variation(%)		
VD	65 °C	7.89	404,568	1845.32	11,843.62	6.42	3.97	1347.64	36.82	72.10	1.96	5851	1.94
	75 °C	7.46	510,217	4935.68	24,881.08	5.04	3.86	1397.08	24.47	112.09	1.69	2315	1.96
	85 °C	7.46	2,663,278	4051.38	83,208.34	20.54	3.86	4134.17	31.09	123.79	3.98	2963	1.96
US-VD	65 °C	7.46	2,045,107	10,869.73	77,556.23	7.13	3.86	3181.58	99.33	204.32	2.06	1113	1.97
	75 °C	7.46	2,593,416	12,330.36	117,452.83	9.53	3.86	4586.38	80.12	240.70	3.00	1030	1.98
	85 °C	7.46	1,248,626	5852.32	48,131.93	8.22	3.86	2244.00	57.90	152.83	2.64	1445	1.95

Note: VD, vacuum drying; US-VD, vacuum drying after ultrasonic pretreatment.

#### 2.6.3 Determination of molecular weight

The molecular weight of each pectin fraction in different dried grapes was determined using the high-performance liquid gel permeation chromatograph method (HPGPC) described by Wang et al. (2013). Briefly, the separation was conducted using an Agilent 1200 HPLC system equipped with a refractive index detector and a TSK-GEL G3000SW_XL_ column (7.5 mm × 300 mm). The chromatographic conditions were as follows: column oven temperature, 25 °C; mobile phase, 0.1 M sodium sulfate solution in PBS (0.01 M, pH 6.8); and flow rate, 0.4 mL/min. Pullulan P-800, P-400, P-200, P-100, P-20, P-10, and P-5 (Showa Denko K. K., Japan) were used as standards for molecular weight measurement (Wang et al., 2013), and the molecular weight was calculated using the standard curve Log*M*_w_ = −0.2419× + 9.6895 (R^2^ = 0.9900).

#### 2.6.4 Fourier transform infrared (FT-IR) spectral analysis

Fourier transform infrared (FT-IR) spectra were recorded with a Nicolet iS50 Spectrometer (Thermos Fisher Scientific, USA). Each pectin fraction in different dried grapes was ground with potassium bromide (KBr) powder and pressed into a pellet for FT-IR spectral measurement in the wavenumber range of 4000 cm^−1^–500 cm^−1^. Background correction with air spectroscopy was required before each scan and the crystal was wiped with 75 % methanol at the end of each measurement.

#### 2.6.5 X-ray diffraction (XRD) analysis

The X-ray diffraction (XRD) patterns of WSP, CSP, and NSP samples were determined by a D2 PHASER X-ray diffractometer (Bruker, Germany). Diffraction measurement was performed with a diffraction angle range of 5° ∼ 65° at a scanning speed of 2°/min.

#### 2.6.6 Circular dichroism (CD) analysis

The circular dichroism (CD) spectrum of each pectin fraction was recorded with a Jasco system (JASCO, Japan) scanning at a wavelength range of 190 nm ∼ 260 nm, a bandwidth of 1 nm, a scanning speed of 1 nm/min, and room temperature of 25 °C. The light chamber of the CD spectrometer was deoxidized with dry nitrogen before use and kept under nitrogen during the experiment. WSP, CSP, and NSP were respectively balanced at baseline with deionized water, 200 mL 0.05 M CDTA solution (containing 0.1 M sodium acetate, pH = 6.5), and 0.05 M sodium carbonate solution (with 0.02 M sodium borohydride). Baseline correction and smoothing were performed prior to spectral analysis of samples.

#### 2.6.7 Microstructure analysis

The microstructure morphology of each pectin fraction was observed using the same scanning electron microscope (SEM) system as in 2.3.2 at an accelerating voltage of 10 kV. Under vacuum conditions, the samples were sputtered with a thin layer of gold, and images were recorded at a magnification of 50 × .

### 2.7 Statistical analysis

Each experiment was performed in triplicate, and data were expressed as means ± standard deviations. Statistical analysis was carried out using the SAS system for Windows V8 (IBM, New York, USA). Significance in comparisons was determined using Tukey's test at a significance level of *P* < 0.05. The Origin 2021 Pro software (Origin Lab, Massachusetts, USA) was used for correlation analysis and PCA. All figures were plotted using PowerPoint 2016 (Microsoft, Washington Redmond, USA) and Origin 2021 Pro.

## 3 Results and discussion

### 3.1 Microstructure of dried grapes

**Fig. 2:**
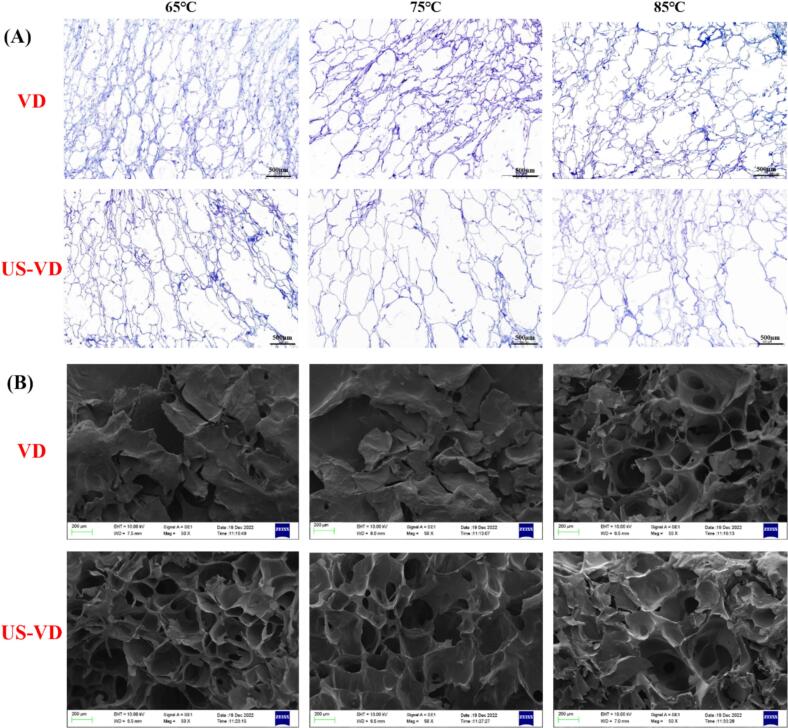
The represent microstructure of different dried grapes by light microscopy (LM, ×3) (A) and scanning electron micrograph (SEM, ×50) (B) (VD, vacuum drying; US-VD, vacuum drying after ultrasonic pretreatment).

The textural properties of dried fruits and vegetables are affected by cell morphology and cell structure (Li et al., 2024). The cell structures of dried grapes after different drying conditions applied in this study are illustrated in Fig. 2(A). Under a microscope, it appeared that grapes dried at 65 °C without ultrasonic pretreatment retained cellular integrity, uniform cell size, and a relatively clear cell wall boundary. With ultrasonic pretreatment, the cell structure became broken and cell size was not uniform. For grapes dried at 75 °C versus those dried at 85 °C, both with and without ultrasonic pretreatment, cell size was more uniform and the cell wall boundary was clearer. These results indicate that the cell structure of dried grapes could be destroyed by ultrasonic pretreatment combined with low drying temperature, but better cell structure might be obtained under higher drying temperatures. During black rosehip fruit drying, drying temperature variations were found to directly influence textural parameters of the final products (Hojjat, Ali, & Ilkay, 2024).

Fig. 2(B) displays SEM images of the various dried grapes. With vacuum drying at 65 °C and 75 °C, severe internal shrinkage and dense structure were observed; corresponding samples with ultrasonic pretreatment showed a good honeycomb and loose porous structure with large and uniform pores. Untreated samples dried at 85 °C demonstrated a better loose honeycomb structure than those of treated grapes. These findings demonstrate that ultrasonic pretreatment combined with lower drying temperatures (65–75 °C) yields a better microstructure in dried grapes, whereas drying at higher temperature (85 °C) can achieve well-structured morphology without pretreatment. This contrasts with Liu et al. (2019), who reported dried jujube cells to be collapsed and shrunken after hot-air drying at 70 °C, particularly at 80 °C. The observed divergence may be attributed to two reasons: Ultrasonication-induced cavitation facilitates porous network formation through cell wall modification at lower drying temperature; Vacuum conditions promote structural preservation via accelerated moisture removal, preventing high-temperature-induced matrix degradation. Notably, no good agreement was obtained between light microscope and SEM observations, potentially due to sampling heterogeneity (i.e., different tissue regions analyzed by each technique).

### 3.2 Cell morphology and porosity of dried grapes

To further understand the pore structure changes in dried grapes, the cell area, cell diameter, number of cells, fractal dimension, and porosity were calculated. Table 1 shows the values obtained for all tested conditions. As the drying temperature increased, the cell area first increased and then decreased, while the cell diameter consistently decreased. After ultrasonic pretreatment, dried grapes exhibited larger cell area and diameter than those without treatment. For drying temperature at 65 °C and 75 °C, the average value of cell area and cell diameter significantly increased. The greatest average cell area (12,330.36 μm^2^) was achieved with US-VD75, while the greatest average cell diameter (99.33 μm) was obtained with US-VD65. These results confirm that drying temperature and ultrasonic pretreatment can change the cell size of dried grapes during vacuum drying. The reason might be that ultrasonic treatment and vacuum environment cause the grapes to form larger cellular pores during drying, especially ultrasonic treated samples. A more uniform cellular structure was obtained due to the formation of microchannels through the US treatment in the lotus root samples (Yu et al., 2024). Regarding cell number, treatments ranked from highest to lowest as follows: VD65 > VD85 > VD75 > US-VD85 > US-VD65 > US-VD75, with a minimum value of 1030 and a maximum value of 5851. This further supports that the cell size of dried grapes increases after ultrasound treatment.

The non-uniformity of cell size in the various treatments was investigated in terms of the coefficient of variation (CV). For without ultrasonic pretreatment samples, the CV of cell area and cell diameter increased with the drying temperature increasing. Ultrasonic-treated dried grapes showed a higher variability parameter than those of untreated samples. These results indicated the destruction of cell structure by ultrasonic and high temperature. Fractal dimension (Df) is a parameter commonly used to describe the structural irregularity of dried fruits and vegetables (Su, Lv, Wang, Wang, & Li, 2020), the more complex the surface structure, the larger the fractal dimension. At the drying temperature of 65 °C and 75 °C, the increase in Df for the US-VD samples (1.966 and 1.976, respectively) compared with the VD samples (1.940 and 1.959) indicated that ultrasonic pretreatment improved the microscopic complexity of dried grapes. However, the US-VD sample exhibited a lower fractal dimension (1.953) than the VD sample (1.959) at 85 °C, meaning that high-temperature drying weaken the cavitation effects induced by ultrasonic pretreatment during vacuum drying.

Porosity is the key to understanding how a crispy texture is obtained in dried food (Yang, Martin, Richardson, & Wu, 2017), especially in determining the sensor quality of dried fruits like crispiness and hardness, etc. (Li et al., 2024). It has been revealed that dried fruits with more and larger pores were crisper. As shown in S-Fig. 2, the porosity of dried grapes increased gradually with drying temperature and was also higher for ultrasonic-treated samples than those untreated. The VD65 samples exhibited the smallest porosity (72.124), and US-VD85 samples were the largest (85.898). The increased porosity in dried materials was linked to diminished mechanical stress and brittleness, as reported by Wang, Liu, Xue, and Li (2020).

### 3.3 Textural characteristics of dried grapes

Texture serves as a critical quality attribute of dehydrated products, with significant implications for consumer preference (Yang et al., 2017). As summarized in Table 2, key textural parameters of dried grapes were quantified through texture profile analysis (TPA).

**Table 2:** Effect of different drying conditions on the texture characteristics of dried grapes.

Drying method	Drying temperature	Parameters				
		Hardness (g)	Rupture distance (mm)	Fracture slope (g/s)	Rupture energy (g·s)	Te
Fresh sample		361.45 ± 25.14^e^	4.26 ± 0.83^a^	89.25 ± 23.40^c^	7.55 ± 1.32^d^	0.25 ± 0.06^e^
US sample		364.00 ± 25.86^e^	5.02 ± 0.90^a^	74.91 ± 13.09^c^	8.87 ± 2.01^d^	0.20 ± 0.04^e^
VD	65 °C	4522.67 ± 241.73^b^	0.65 ± 0.03^de^	2949.64 ± 156.97^ab^	1232.03 ± 59.99^b^	0.59 ± 0.05^b^
	75 °C	6579.33 ± 374.57^a^	0.71 ± 0.02^cd^	2090.63 ± 182.89^b^	1065.40 ± 128.05^bc^	0.27 ± 0.04^d^
	85 °C	3322.67 ± 80.83^d^	0.52 ± 0.03^d^	1002.02 ± 118.39^b^	277.33 ± 60.25^c^	0.29 ± 0.04^d^
US-VD	65 °C	3399.00 ± 238.16^d^	0.56 ± 0.04^e^	2423.60 ± 358.13^ab^	745.19 ± 54.55^c^	0.68 ± 0.08^a^
	75 °C	3695.00 ± 283.67^cd^	0.80 ± 0.02^b^	2595.17 ± 578.41^ab^	1716.20 ± 193.75^a^	0.48 ± 0.03^c^
	85 °C	4075.33 ± 101.53^c^	0.72 ± 0.15^cd^	3337.08 ± 1000.86^a^	1679.55 ± 420.72^a^	0.67 ± 0.02^ab^

Note: US, ultrasonic pretreatment; VD, vacuum drying; US-VD, vacuum drying after ultrasonic pretreatment. Te, Texture characteristic index. Different letters in the same column indicate significant differences at *P*<0.05.

There was no significant difference in hardness and brittleness between fresh and US grapes (*P* >0.05). After vacuum drying, the hardness and brittleness of dried grapes increased significantly (*P* < 0.05). The highest hardness (6579.33 g) was obtained for the sample without ultrasonic pretreatment during vacuum drying at 75 °C, followed by VD65 (4522.67 g) and VD85 (3322.67 g). Notably, although sample VD75 had higher porosity than VD65 (S-Fig. 2), severe shrinkage and a dense microstructure observed in VD75 (Fig. 2B) also led to its highest hardness. This is not consistent with the research of Liu et al. (2021), who found there was a positive correlation between high porosity and lower hardness in puffed peach chips. In contrast to VD samples, the decreased hardness of US-VD samples dried at 65 °C and 75 °C indicated that ultrasonic processing reduced the hardness of dried grapes. This is consistent with the findings of Yildiz and Izli (2019), who reported that untreated samples exhibited a more denser porous structure with higher hardness than ultrasonic-treated samples. However, the hardness of the US-VD85 (4075.33 g) sample was higher than that of VD85 (3322.67 g) dried grapes, illustrating that the opposite results might be obtained for ultrasonic pretreatment samples during high-temperature drying and different drying methods.

Meantime, for US-VD samples, the higher the drying temperature, the higher the hardness, which might be associated with the degradation of cell wall constituents of dried grapes, including cell wall polysaccharides. For samples without ultrasonic pretreatment, the fracture slope and rupture energy decreased with increasing drying temperature, while the US-VD samples showed an opposite trend. This indicated that some brittleness indicators could be improved by ultrasonic pretreatment. The largest rupture energy and rupture distance were observed with US-VD75, at 1716.20 g·s and 0.80 mm respectively.

As a comprehensive indicator for crispy fruits, a higher Te value indicates a better texture quality. Compared with fresh and US grapes, the Te value increased significantly for dried samples (*P* < 0.05) (Table 2), meaning that drying can improve the texture of grapes. As the drying temperature increasing, the Te value decreased significantly (*P*< 0.05) for VD samples, while the Te value first decreased then increased for US-VD samples. The results indicated that high temperature was not conducive to the formation of good texture in dried grapes.

As shown in Table 2, dried grapes with ultrasonic pretreatment showed better Te values than those without ultrasonic-treated samples. The Te value of US-VD65 was the highest (0.68), followed by US-VD85 (0.67), VD65 (0.59), US-VD75(0.48), VD75 and VD85. Meanwhile, there was no significant difference between VD75 and VD85 samples (*P* > 0.05). These results suggest that low-frequency ultrasonic optimized the pore structure due to the formation of microchannels during low-temperature drying (Fig. 2B), thus improving the texture properties. Several studies have reported that ultrasonic pretreatment improves the texture quality of dried sweet potato slices (Wu, Zhang, Ye, & Yu, 2020) and jackfruit (Wu, Guo, Guo, Ma, & Zhou, 2021). The reason might be that the sound waves and cavitation effect destroys cell micro-channels, leading to better texture of the final products.

From above results we obtained that drying temperature was the main factor affecting texture quality of untreated dried grapes. It was worth noting that ultrasonic pretreatment could reduce the influence of temperature and improve the texture of dried grapes.

### 3.4 Variation of pectin composition in dried grapes

#### 3.4.1 Galacturonic acid(GalA) content of three pectin fractions

Pectin is a branched polysaccharide predominantly composed of galacturonic acid (GalA) as its backbone, with diverse monosaccharide residues covalently attached through heterogeneous linkages to generate complex side-chain structures. Hence, GalA can be used to indicate the content change of pectin. The results of the GalA content changes of three pectin fractions in different grape samples are shown in Table 3.

**Table 3:** Effect of different drying conditions on GalA content, monosaccharide composition and structure characteristics of three pectin fractions in dried grapes.

	Drying method	Drying temperature	Parameters										
			GalA(mg/g AIR)	Gal(mg/g AIR)	Ara(mg/g AIR)	Rha(mg/g AIR)	Man(mg/g AIR)	GluA(mg/g AIR)	Glu(mg/g AIR)	Ratio1 (%)	Ratio2 (%)	Ratio3 (%)	Mw (KDa)
WSP	Fresh sample		23.19 ± 4.25^bc^	2.80 ± 0.03^a^	0.43 ± 0.07^c^	1.54 ± 0.20^a^	0.49 ± 0.03^f^	0.33 ± 0.04^a^	0.19 ± 0.03^b^	4.86	0.066	2.10	8.28 ± 0.38^a^
	US sample		21.04 ± 2.58^c^	1.63 ± 0.01^e^	0.20 ± 0.03^g^	0.98 ± 0.03^b^	0.54 ± 0.01^e^	0.29 ± 0.01^c^	0.10 ± 0.02^c^	7.49	0.047	1.87	8.23 ± 0.02^a^
	VD	65 °C	23.85 ± 1.63^bc^	2.34 ± 0.08^c^	0.37 ± 0.01^d^	0.92 ± 0.00^d^	0.53 ± 0.02^e^	0.18 ± 0.02^g^	0.22 ± 0.01^a^	6.57	0.038	2.95	7.30 ± 0.03^f^
		75 °C	32.53 ± 0.14^a^	2.37 ± 0.03^b^	0.62 ± 0.03^a^	0.75 ± 0.02^e^	0.67 ± 0.04^b^	0.26 ± 0.05^e^	0.12 ± 0.00^c^	8.69	0.023	3.99	7.45 ± 0.07^e^
		85 °C	25.66 ± 2.19^b^	2.33 ± 0.01^c^	0.34 ± 0.06^e^	0.66 ± 0.12^f^	0.52 ± 0.02^e^	0.27 ± 0.01^d^	0.06 ± 0.01^d^	7.71	0.025	4.05	7.72 ± 0.01^d^
	US-VD	65 °C	16.73 ± 1.39^d^	0.86 ± 0.04^g^	0.37 ± 0.01^d^	0.93 ± 0.06^c^	0.62 ± 0.01^c^	0.19 ± 0.02^f^	0.23 ± 0.02^a^	7.75	0.056	1.32	7.78 ± 0.03^c^
		75 °C	23.56 ± 2.42^bc^	1.65 ± 0.09^d^	0.52 ± 0.03^b^	0.67 ± 0.09^f^	0.69 ± 0.01^a^	0.30 ± 0.06^b^	0.11 ± 0.04^c^	8.30	0.028	3.24	8.26 ± 0.03^a^
		85 °C	22.55 ± 1.37^bc^	1.12 ± 0.46^f^	0.25 ± 0.02^f^	0.66 ± 0.01^f^	0.56 ± 0.04^d^	0.33 ± 0.15^a^	0.10 ± 0.02^c^	11.11	0.029	2.08	7.92 ± 0.08^b^
CSP	Fresh sample		1.14 ± 0.02^e^	0.49 ± 0.07^c^	0.36 ± 0.01^c^	0.62 ± 0.00^b^		0.42 ± 0.15^a^		0.78	0.543	1.37	8.22 ± 0.06^d^
	US sample		4.07 ± 0.46^a^	0.49 ± 0.01^c^	0.35 ± 0.02^d^	0.60 ± 0.01^c^		0.40 ± 0.01^b^		2.83	0.147	1.40	8.38 ± 0.00^c^
	VD	65 °C	1.92 ± 0.07^d^	0.45 ± 0.03^e^	0.24 ± 0.03^e^	0.53 ± 0.01^d^	/	0.36 ± 0.10^d^	/	1.57	0.276	1.30	8.45 ± 0.03^b^
		75 °C	1.04 ± 0.24^e^	0.63 ± 0.02^b^	0.45 ± 0.04^b^	0.62 ± 0.03^b^	/	0.41 ± 0.01^b^	/	0.61	0.596	1.74	8.14 ± 0.04^e^
		85 °C	2.87 ± 0.07^b^	0.42 ± 0.00^g^	0.23 ± 0.06^e^	0.53 ± 0.03^d^	/	0.33 ± 0.06^e^	/	2.43	0.185	1.23	7.58 ± 0.03^f^
	US-VD	65 °C	0.85 ± 0.05^f^	0.46 ± 0.03^d^	0.24 ± 0.02^e^	0.54 ± 0.02^d^	/	0.38 ± 0.02^c^	/	0.69	0.645	1.29	8.89 ± 0.52^a^
		75 °C	2.43 ± 0.03^c^	0.70 ± 0.09^a^	0.47 ± 0.03^a^	0.64 ± 0.01^a^	/	0.41 ± 0.01^b^	/	1.34	0.263	1.83	7.49 ± 0.03^g^
		85 °C	0.80 ± 0.07^f^	0.44 ± 0.03^f^	0.15 ± 0.04^f^	0.53 ± 0.01^d^	/	0.26 ± 0.06^f^	/	0.71	0.663	1.11	7.56 ± 0.02^f^
NSP	Fresh sample		9.83 ± 0.02^b^	0.47 ± 0.03^a^	1.59 ± 0.07^a^	1.60 ± 0.08^c^		0.18 ± 0.15^c^	0.39 ± 0.01^a^	2.69	0.163	1.29	8.46 ± 0.02^c^
	US sample		11.97 ± 1.71^a^	0.41 ± 0.01^d^	1.46 ± 0.01^b^	1.62 ± 0.03^b^		0.16 ± 0.01^d^	0.36 ± 0.00^c^	3.43	0.135	1.15	8.04 ± 0.02^g^
	VD	65 °C	12.11 ± 0.46^a^	0.42 ± 0.00^c^	1.39 ± 0.13^g^	0.93 ± 0.07^e^	/	0.14 ± 0.10^e^	0.27 ± 0.02^f^	4.42	0.077	1.95	8.76 ± 0.02^a^
		75 °C	3.44 ± 0.07^e^	0.46 ± 0.01^b^	1.43 ± 0.03^d^	1.62 ± 0.03^b^	/	0.20 ± 0.00^b^	0.36 ± 0.00^c^	0.98	0.471	1.17	8.23 ± 0.01^d^
		85 °C	4.43 ± 0.07^d^	0.36 ± 0.02^f^	1.41 ± 0.02^f^	0.82 ± 0.03^f^	/	0.13 ± 0.00^f^	0.33 ± 0.18^d^	1.71	0.185	2.16	8.19 ± 0.02^e^
	US-VD	65 °C	7.78 ± 0.44^c^	0.39 ± 0.01^e^	1.42 ± 0.02^e^	0.94 ± 0.02^d^	/	0.13 ± 0.01^f^	0.28 ± 0.00^e^	2.85	0.118	1.97	8.51 ± 0.03^b^
		75 °C	5.43 ± 0.19^d^	0.48 ± 0.00^a^	1.45 ± 0.03^c^	1.65 ± 0.01^a^	/	0.24 ± 0.01^a^	0.38 ± 0.02^b^	1.52	0.304	1.17	8.08 ± 0.01^g^
		85 °C	3.78 ± 0.44^e^	0.32 ± 0.01^g^	1.40 ± 0.03^f^	0.72 ± 0.01^g^	/	0.10 ± 0.01^g^	0.35 ± 0.00^c^	1.55	0.190	2.39	8.11 ± 0.01^f^

Note: US, ultrasonic pretreatment; VD, vacuum drying; US-VD, vacuum drying after ultrasonic pretreatment. GalA: Galacturonic acid content; Gal: Galactose; Ara: Arabinose; Rha: Rhamnose; Man: Mannose; GluA: Glucuronic acid; Glu: Glucose. Ratio 1, GalA/(Fuc + Rha + Ara + Gal+Xyl), represents the linearity of pectin; Ratio 2, Rha/GalA, represents the contribution of RG to the pectin population; Ratio 3, (Ara + Gal)/Rha, represents the length of side chains attached to RG-I. ‘/’ represents no detected, and different letters for each pectin fraction in same column indicate significant differences at *P*<0.05.

For different grape samples, WSP accounted for the greatest fraction (16.73 mg/g AIR-32.53 mg/g AIR) while CSP comprised the least (0.80 mg/g AIR-4.07 mg/g AIR), demonstrating that the majority of pectin exists as homogalacturonan (HG) macromolecules, while WSP displayed significantly higher galacturonic acid abundance and backbone linearity compared to other fractions. This result differs from the findings of Wang et al. (2018), who identified NSP as the dominant fraction in grapes. As shown in Table 3, compared with fresh samples, ultrasonic pretreatment led to the decrease of WSP content (23.19 to 21.04 mg/g AIR), the increased of CSP (1.14 to 4.07 mg/g AIR) and NSP (9.83 to 11.97 mg/g AIR) content in US samples. However, the increased content of WSP was obtained in carrots after ultrasonic pretreatment (Guo, Wu, et al., 2022). This might be different raw materials have the different sensitivities to ultrasonic.

Under vacuum drying alone, increasing the temperature from 65 °C to 75 °C elevated water-soluble pectin (WSP) content, while cellulose-bound pectin (CSP) and non-soluble pectin (NSP) fractions decreased proportionally (Table 3). This might be because of the thermal degradation mechanisms, including thermosolubilisation of matrix polysaccharides, β-elimination, and depolymerization of pectin backbones (Imaizumi et al., 2017). Notably, the observed WSP accumulation likely resulted from the cleavage of ionic bonds bridging CSP/NSP to the cell wall network, as described by Guo and Wu et al. (2022), who demonstrated heat-induced interconversion between pectin fractions. Specifically, covalent and ionic linkages anchoring pectin to structural polymers were disrupted at 75 °C, generating loosely bound pectin fragments. This structural disorganization may explain the increased hardness in these samples (Table 2), contrasting with the report of Guo et al. (2022) where high WSP in thermosonicated carrot tissue (70 °C) correlated with reduced hardness.

With vacuum drying at 85 °C, we obtained a very different result, with no significant change in WSP content (*P* > 0.05), whereas CSP content increased significantly (*P* < 0.05) and NSP content decreased correspondingly (*P*<0.05) (Table 3). Together, these results show that interconversion among the pectin fractions varies with different drying temperatures.

With the use of ultrasonic pretreatment, WSP, CSP, and NSP contents were consistently lower than those of untreated samples. This was mainly due to beta-elimination and depolymerization of matrix polysaccharides during ultrasonic pretreatment. It was worth noting that the higher NSP and CSP contents was detected in US-VD75 samples than VD75 grapes. According to Gao et al. (2024), the part of WSP was converted into CSP with stronger chelating ability, resulting in a significant increase in the content of CSP under the US-VD75 drying condition. This result might be the reason that lower Te value for US-VD75 samples than that of grapes vacuum drying at 65 and 85 °C after ultrasonic pretreatment (Table 2). Therefore, these findings support our hypothesis that the variation in pectin content is related to the texture properties of dried grapes.

#### 3.4.2 Monosaccharide composition and sugar ratio analysis

Changes in pectin chain conformation could be predicted from the monosaccharide composition and content of cell wall polysaccharides (Qiu et al., 2019). Table 3 gives the observed monosaccharide compositions of the three pectin fractions in each grape samples. Due to the relatively low content of certain monosaccharides, this study primarily focused on analyzing five monosaccharides and one uronic acid. For dried samples with or without ultrasonic pretreatment, the highest total pectin monosaccharide content (GalA + Gal + Ara + Rha + Man + GluA+ Glu) was obtained for VD75 (47.98 mg/g AIR), followed by VD65 (47.17 mg/g AIR), US-VD75 (41.78 mg/g AIR), VD85 (41.70 mg/g AIR), US-VD85 (33.42 mg/g AIR), and finally US-VD65 (33.34 mg/g AIR). This ranking suggests that pectin composition and structure might be destroyed by ultrasonic and high temperature. Considering each fraction separately, the highest monosaccharide content was observed in WSP (19.93 mg/g AIR - 37.32 mg/g AIR), followed by NSP (6.67 mg/g AIR - 15.26 mg/g AIR) and then CSP (2.18 mg/g AIR - 4.65 mg/g AIR). This finding is consistent with the pattern reported for three pectin fractions in dried apple slices (Yu et al., 2023).

Concerning individual monosaccharides, the proportion of GalA relates to the HG domain of the pectin backbone (Yang, Mu, & Ma, 2018). GalA and Rha comprise the main components of the backbone of RG-I, while Gal and Ara are constituents of the side chain (galactan, arabinan, and arabinogalactan), and GluA and Man usually occur in side chains of RG-II (Mohnen, 2008). For different dried grapes, the changes in monosaccharides content may reflect the changes in sugar ratio and thereby affect the structure of three pectin fractions.

Ratio 1 represents the linearity of pectin and is related to the HG domain. In VD and US-VD samples, the value of ratio 1 was higher for WSP than for CSP or NSP, indicating the WSP fraction to have higher linearity, which is consistent with the results in Section 3.4.1. Notably, ratio 1 of the WSP increased with the drying temperature increasing, inconsistent with the finding of Yu et al. (2023) that thermal treatment could break the linear structure of the WSP fraction in dried apple slices. It means that the structure of WSP in apple and grape tissues is differently affected by thermal processing. For US-VD75 samples, the linearity of WSP decreased, while the linearity of CSP and NSP increased to varying degrees, suggesting the backbone of WSP molecules was broken during US-VD at 75 °C, which resulted in its conversion to CSP and NSP. Gao et al. (2024) reported that ultrasonic combined with infrared drying could decrease the linearity of WSP in carrots, while increasing the linearity of CSP and NSP. However, the linearity of WSP increased, and the linearity of CSP and NSP decreased under US-VD at 65 °C and 85 °C. Combined with the texture results in Table 2, it might be the high linearity of WSP led to a crisper texture for US-VD at 65 °C and 85 °C samples.

Ratio 2 indicates the contribution of the RG-I domain in pectin. As stated by Yu et al. (2023), the RG-I region is the predominant structure in a pectin fraction when the value of ratio 2 ranges from 0.05 to 1. Given the Gal, Ara, and Rha levels obtained for the NSP and CSP fractions in dried grapes, the RG-I region should be the main structure of the two pectin fractions. In both VD75 and US-VD75 samples, Gal and Ara content increased significantly in the CSP and NSP fractions; moreover, the ultrasonic-treated samples contained more Gal and Ara than that of untreated. These results indicate that thermal processing and ultrasonic pretreatment could destroy the side chain structure of the RG-I region, leading to the observed increase of Gal and Ara content. Mohnen (2008) reported that the arabinan side chain of RG-I might determine the flexibility and extensibility of plant cell walls, and its degradation was related to changes in tissue hardness. This might help explain the poor texture of the VD75 and US-VD75 samples (Table 2).

Ratio 3 indicates the branching extent of the RG-I region. For the WSP fraction, it had the highest degree of branching, and ratio 3 increased with drying temperature increasing, indicating thermal processing to induce a more branched RG-I domain. Ultrasonic-treated samples had higher ratio 3 values in WSP than those of vacuum dried alone, revealing that ultrasonic pretreatment promotes further branching of the RG-I domain. In addition, the WSP fraction was high in Gal, Rha, Man, Ara, and GluA. As shown in Table 3, the sugar ratio 3 of VD65, US-VD65 and US-VD85 was all less than 3.00, while the three dried grapes had the better texture (Table 2). This result indicates that the lower branching degree of WSP RG-I region could improve the textural property of dried grapes. For the NSP fraction, the highest ratio 3 value was observed in US-VD85, followed by VD85, US-VD65, VD65, then US-VD75, and VD75. These results indicate the RG-I region of NSP in dried grapes to develops a small degree of branching under low-temperature drying (≤ 75 °C). In contrast, ratio 3 of the CSP fraction was elevated in the US-VD75 and VD75 samples compared to others.

#### 3.4.3 Molecular weight analysis

**Fig. 3:**
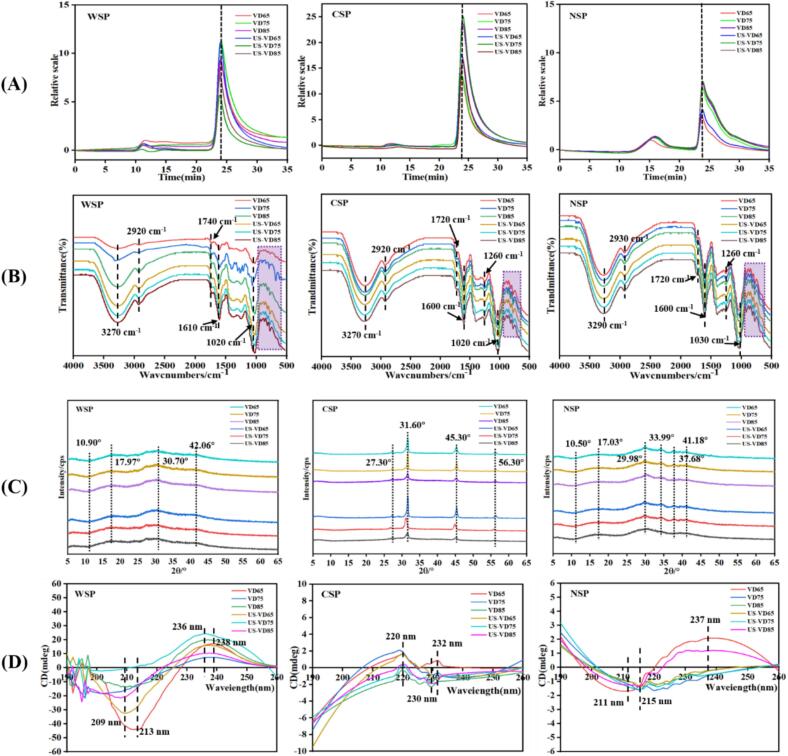
The molecular weight distribution(A), FT-IR spectrum(B), XRD curve(C) and Circular dichroism (D)of three pectin fractions in different dried grapes.

Molecular weight (Mw) is a structural characteristic, the changes in polysaccharide structure of pectin are reflected in its Mw. Fig. 3(A) presents the chromatograms obtained for the three pectin fractions using high-performance size exclusion chromatography (HPSEC). It is evident in these chromatograms that the WSP and CSP fractions each have one obvious polysaccharide component, while NSP contains two macromolecular polymers. This finding differs from a prior study of dried okra pectin (Xu et al., 2021). There were no obvious changes in high-molecular-weight pectin polymers for different grape samples (Table 3). In VD samples, the weights of three pectin fractions ranked as follows: NSP (8.19 kDa–8.76 kDa) > CSP (7.58 kDa–8.45 kDa) > WSP (7.30 kDa–7.72 kDa). In general, as drying temperature increased, the Mw of WSP increased while that of NSP and CSP decreased. This result is consistent with the report of Yu et al. (2023), who found the Mw of WSP in hot-air drying (HAD) 60 and HAD 90 apple slices to increase significantly and that of NSP and CSP to decrease slightly. The authors attributed this phenomenon to cellular wall substances releasing more pectin during higher-temperature thermal processing, which then further degraded into WSP in the final dried fruits (Yu et al., 2023). Comparing US-VD to VD samples at the same temperature, the Mw of WSP increased significantly (*P* < 0.05) while CSP and NSP exhibited a decreasing trend. Thus, ultrasonic pretreatment could further reduce the Mw of CSP and NSP and increase that of WSP. Therefore, high-temperature drying and ultrasonic pretreatment could reduce the Mw of ionically and covalently cross-linked pectin fractions and increase the Mw of loosely-bonded pectin. Xu et al. (2021) reported that when pectin is dried at above 20 °C and okra is dried by vacuum pulsation drying (VPD) or HAD at 60 °C, the Mw of pectin polysaccharide is decreased. According to Sila, Smout, Elliot, Loey, and Hendrickx (2006), the larger the molecular weight of the pectin, the more significant its cross-linking effect.

#### 3.4.4 FT-IR analysis

The main functional groups and chemical bonds of three pectin fractions in dried grapes were analyzed by FT-IR in the wavenumber region of 500–4000 cm^−1^ (Fig. 3(B)). All three fractions exhibited a strong and wide peak between 3000 cm^−1^ and 3500 cm^−1^, which represented the stretching vibration of hydroxyl groups (O—H) due to the inter- and intra-molecular hydrogen bonding of GalA in pectins. Another common peak at 2800 cm^−1^ to 3000 cm^−1^ was caused by stretching and bending vibrations of C—H groups (CH, CH2, and CH3) (Romdhane et al., 2017). Peaks from O—H and C—H groups became especially intense in the WSP fraction of VD85 and all US-VD samples, indicating that the greater freedom of O—H and C—H was obtained in WSP after high-temperature processing and ultrasonic pretreatment. The band at about 1720 cm^−1^ and 1740 cm^−1^ corresponded to the C=O stretching vibration of ester carbonyl groups, while a sharp peak at about 1600 cm^−1^ was attributed to the stretching of carboxylate ions (COO-) (Kong et al., 2015). The ratio of the peak area at 1740 cm^−1^ to the sum of the peak areas at 1740 cm^−1^ and 1600 cm^−1^ represented the degree of esterification (DE) in the pectin (Yu et al., 2023). For WSP fraction of VD75, VD85, and all US-VD samples, the peak at 1600 cm^−1^ became intense, meaning that higher drying temperature reduced the DE, especially in conjunction with ultrasonic pretreatment. In contrast, the CSP and NSP fractions did not exhibit significant differences in the 1600 cm^−1^ peak for any treatment. These results indicate that β-elimination occurred more easily in the WSP fraction, especially under ultrasonic-assisted high-temperature vacuum drying. Another notable absorption band occurred at about 1020 cm^−1^ in three pectin fractions, indicating the presence of pyranose and furanose (Lin et al., 2021). This peak became intense in the WSP of VD85 and all US-VD samples. Finally, there were several notable peaks at 1000 cm^−1^–600 cm^−1^ (light purple shaded area, Fig. 3(B)), which is considered the characteristic fingerprint region of pectin. Overall, the molecular structure of the WSP fraction was most impacted by processing, especially after vacuum drying at 85 °C and ultrasonic-assisted vacuum drying at any temperature. This processing mainly affected the hydrogen bond structure.

#### 3.4.5 X-ray analysis

X-ray diffraction refers to a phenomenon that occurs when X-rays pass through a crystal and can be used to determine whether a substance is amorphous or crystalline. As shown in Fig. 3(C), the WSP and NSP fractions obtained from the various dried grape samples had similar XRD patterns. Both exhibited typical diffraction peaks at about 10°, 17°, 30°, and 42°, and those peaks were not sharp and strong, which indicated that WSP and NSP have typical amorphous polymer structures. The CSP fractions showed diffraction peaks at 27.30°, 31.60°, 45.30°, and 56.30°, of which the peaks at 31.60° and 45.30° were intense and sharp, suggesting this fraction to have a crystalline polymer structure. Moreover, in samples with ultrasonic pretreatment, these crystalline signals showed an obvious left-shifting trend and were weaker relative to corresponding samples without ultrasonic-treated, indicating the ultrasonic pretreatment changed CSP crystallinity. The reduction of peak intensity probably resulted from the breakdown of hydrogen bonds in the polysaccharide polymer network (Wu et al., 2020). Compared with strong and rigid ordered crystals, amorphous polymers have an elastic structure (Xu et al., 2021) and lower-magnitude intermolecular forces (Ye, Li, & Zhao, 2020). Taken together, these results confirmed that WSP and NSP fractions in dried grapes are more unstable in structure than that of CSP fraction, consistent with the results observed for pectin content, monosaccharide content, and molecular weight.

#### 3.4.6 CD analysis

Circular dichroism is able to directly evaluate conformational transformations of polysaccharides containing uronic acid, carboxylic acid groups, and amide chromophores. Pectin polysaccharides have a positive response near the circular dichroism wavelength of 210 nm because of the migration of carboxyl (COO-) groups, such as in the n → π* transition, on the pectin molecular chain. Plaschina, Braudo, and Tolstoguzov (1978) previously showed that inter-molecular interactions might have a great effect on the optical activity of carboxyl chromophores. Fig. 3(D) presents the circular dichroic spectra of the three pectin fractions from various dried grape samples investigated in this study.

The WSP fraction showed an obvious inverted peak near 210 nm and a positive response value in the range of 230–240 nm. These results are consistent with the study of Xu et al. (2021), who reported an inverted peak at 200–210 nm and a positive peak at 220–230 nm for okra pectin molecules treated with hot air drying and vacuum pulse drying. In VD samples, the positive peak at 238 nm shifted to the left with drying temperature increasing, and the inverted peak near 210 nm disappeared. Among US-VD samples, the peak at 213 nm and 238 nm shifted to the left and became weak for ultrasonic-assisted drying at 65 °C and 85 °C, while the peak near 210 nm disappeared and that at 236 nm became strongly intense with drying at 75 °C. Signal intensity is related to ellipticity, the stronger the signal intensity, the larger the ellipticity and the tighter the structure (Plaschina et al., 1978). Thus, these peak changes indicate that drying temperature and ultrasonic pretreatment greatly impact the structure and chain conformation of WSP.

Comparatively, the circular dichroism spectra of both CSP and NSP fractions showed weak signal intensity. For NSP, a negative absorption peak was found at 210–220 nm, which shifted to the right with increasing drying temperature and the use of ultrasonic pretreatment. In addition, a positive peak was found near 237 nm in the VD65 and US-VD85 samples. For CSP, there was no positive response near 210 nm, while a positive peak was found near 220 nm. Relative to the US-VD65 sample, all other treatment conditions impacted the peak near 220 nm. CSP fractions also exhibited a peak near 230 nm, which became inverted and shifted to the left with higher temperature processing and ultrasonic pretreatment. All told, these results support that high temperature and ultrasonic pretreatment might influence the structure and chain conformation of NSP and CSP. Xu et al. (2021) found that ultrasonic pretreatment of okra pectin could significantly reduce ellipticity by decreasing the degree of cross-linking during heat processing. However, the reason of the relationship between signal intensity and ellipticity was unclear.

#### 3.4.7 Microstructure analysis

S-Fig. 3 shows SEM images of the three pectin fractions after VD and US-VD. A different morphological structure is evident for each samples, supporting that thermal processing and ultrasonic pretreatment could change the micromorphology of WSP, CSP, and NSP in dried grapes. Under VD at 65 °C, the surface of WSP appeared flocculent and had the structure of a large, curling sheet, that of CSP was spheroidal and ramified, and the NSP surface harbored coarse rod-like substances. With high-temperature processing, the sheet structure of WSP disappeared, no spheroidal particulates or filament-like connections were observed on CSP, and few of the rod-like substances remained on NSP. Meanwhile, with US-VD at 65 °C, WSP appeared as a dense flocculent structure, CSP had many spherical particles, and NSP showed small, clumpy, rod-like structures on its surface. With increasing drying temperature, WSP took on a rough layered lamellar surface, the spherical particles of CSP were replaced by a dense structure, and the surface of NSP became smooth. These substantial differences in morphological features and microstructures across the different treatments might be associated with the above-mentioned structure and chain conformation changes induced by high temperature and ultrasonic pretreatment.

### 3.5 Correlation analysis

**Fig. 4:**
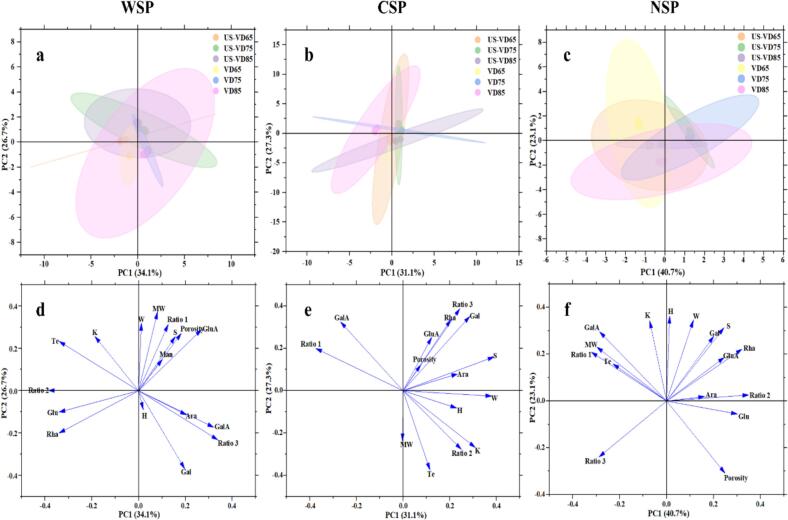
Score and loading plot of the first two components from the Principal Component Analysis of WSP, CSP and NSP (a, b and c represent the score plot of WSP, CSP and NSP, respectively; d, e and f represent the loading plot of WSP, CSP and NSP, respectively).

PCA and correlation analysis were conducted to clarify the effect of pectin characteristics on the textural properties of dried grapes. Fig. 4 depicts the score and loading plots of the first two principal components from the PCA. The distances between samples on the score chart indicate how similar they are in product attributes (Yang et al., 2016), while for indexes, the distance from the zero point in the loading plot indicates their correlation with the principal components (Bendini et al., 2011). The PCA results indicated the three pectin fractions each contribute differently to the texture quality of dried grapes.

For WSP (Fig. 4a), the respective contribution rates of PC1 and PC2 were 34.1 % and 26.7 %. PC2 was negatively correlated with VD samples, but positively with US-VD samples. PC1 was positively correlated with sugar ratio 3 and the GalA, Gal, and Ara contents, while negatively with sugar ratio 2 and the Rha and Glu contents. Meanwhile, sugar ratio 1 and molecular weight of WSP both contributed significantly to PC2. For CSP (Fig. 4b), the contribution rates of the first two principal components were 31.1 % and 27.3 %, respectively. All samples were similar to each other except VD85. For NSP (Fig. 4c), the respective contribution rates of PC1 and PC2 were 40.7 % and 23.1 %. VD75 samples with and without ultrasonic pretreatment were similar, and US-VD65 and US-VD85 also grouped together, while the other two drying conditions had distinct characteristics.

**Fig. 5:**
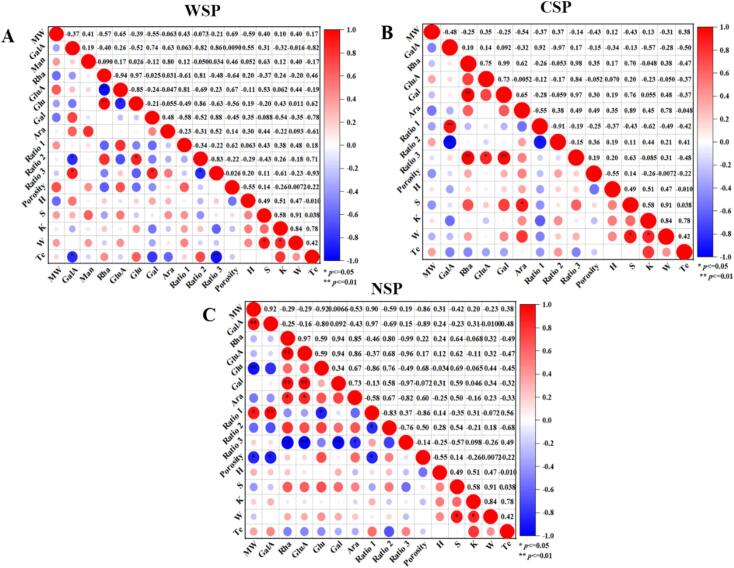
The correlation between pectin characteristic parameters and the texture characteristic factors of three pectin fractions (The blue dots represent the negative correlation, the red dots represent the positive correlation, and the size of the dots and the depth of the color represent the size of the correlation coefficient.). (For interpretation of the references to color in this figure legend, the reader is referred to the web version of this article.)

Fig. 5 shows the correlation of the three pectin fractions with the textural properties of dried grapes. For WSP, the GalA content of WSP was negatively correlated with the Te value (*P* < 0.05) (Fig. 5A), indicating that low-linear pectin may improve crispness by weakening the cell wall rigidity, which was consistent with the high Te value of US-VD65. And Te value showed a highly significantly negative correlation with sugar ratio 3 (*P* < 0.01), which suggested that less degree of RG-I branching in WSP could give a better texture quality for dried grapes, this was consistent with the results of mono analysis in 3.4.2. The GalA content, and sugar ratio 1 of NSP was significantly negatively correlated with the porosity of dried grapes (*P* < 0.05) (Fig. 5B), illustrating that high-linear pectin can destroy the microstructure by influencing the cell area, which was in line with the low porosity of VD65. For CSP (Fig. 5C), Ara content was positively correlated with breaking distance (*P* < 0.05), which suggested that CSP might have an effect on the texture quality of dried grapes, but did not play a dominant role.

## 4 Conclusion

This study investigated the effects of ultrasonic pretreatment and different vacuum drying temperature on dried grapes. Using untreated samples as controls, the microstructure, porosity, textural properties, and attributes of WSP, CSP, and NSP in dried grapes were analyzed. Results showed that the US-VD65 drying condition produced optimal pore structure, brittleness, and Te value for dried grapes. Rising temperatures increased the proportions of WSP and CSP, reducing intercellular adhesion and increasing the hardness of dried grapes. Ultrasonic pretreatment induced the conversion of WSP to CSP and NSP during vacuum drying at 75 °C. Ultrasonic pretreatment and different drying temperature could modify the GalA content, monosaccharide composition, molecular weight and microstructure of WSP, CSP and NSP in grapes. The GalA content and ratio 3 of WSP were the main factors affecting the brittleness and Te value of dried grapes. The GalA content changes of NSP could regulate the porosity of dried grapes. These foundings highlight the combined effect of ultrasonic pretreatment and drying temperature control in optimizing fruit texture properties (microstructure, porosity, crispness, etc.), providing critical insights for regulating the textural quality of crispy fruit products. And the US-VD65 process can be used as an efficient low - energy - consumption drying solution for the production of grapes with high crispness.

## CRediT authorship contribution statement

**Yadong Xiao:** Writing – review & editing, Writing – original draft, Methodology, Investigation, Formal analysis, Data curation, Conceptualization. **Yiwen Yang:** Writing – original draft, Software, Methodology. **Yayuan Xu:** Writing – review & editing, Software. **Lei Feng:** Writing – review & editing, Methodology. **Meimei Nie:** Methodology, Investigation. **Liying Niu:** Writing – review & editing, Funding acquisition. **Chunquan Liu:** Writing – review & editing. **Chunju Liu:** Writing – review & editing. **Dajing Li:** Writing – review & editing, Supervision, Funding acquisition, Conceptualization. **Zhifang Yu:** Writing – review & editing, Supervision, Conceptualization.

## Declaration of competing interest

The authors declare that they have no known competing financial interests or personal relationships that could have appeared to influence the work reported in this paper.

## Data Availability

Data will be made available on request.

## Appendix A Supplementary data

Supplementary material 1 Image 1

Supplementary material 2 Image 2

Supplementary material 3 Image 3

Supplementary material 4 Image 4
