# Supplementary material for: Ultrasonic pretreatment and drying temperature-induced modifications of three pectin fractions affect the microstructure and textural properties of dried grapes
Source: Food Chem X. 2025 Jun 4;28:102633. doi: 10.1016/j.fochx.2025.102633 (PMC12173666; doi:10.1016/j.fochx.2025.102633)
Supplement: Supplementary file 1 — Supplementary material 1 [file mmc1.docx]

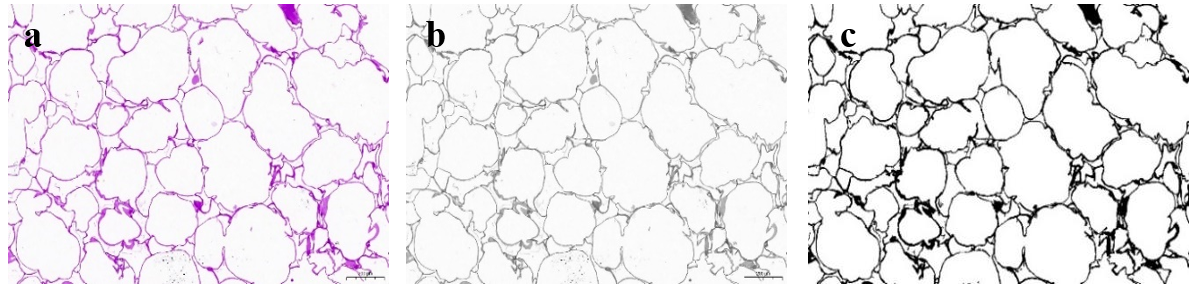


Stained light microscope image Gray scale image Binarization processing

S-Fig. 1. Process of sample light microscope diagram
