# Supplementary material for: Ultrasonic pretreatment and drying temperature-induced modifications of three pectin fractions affect the microstructure and textural properties of dried grapes
Source: Food Chem X. 2025 Jun 4;28:102633. doi: 10.1016/j.fochx.2025.102633 (PMC12173666; doi:10.1016/j.fochx.2025.102633)
Supplement: Supplementary file 2 — Supplementary material 2 [file mmc2.docx]

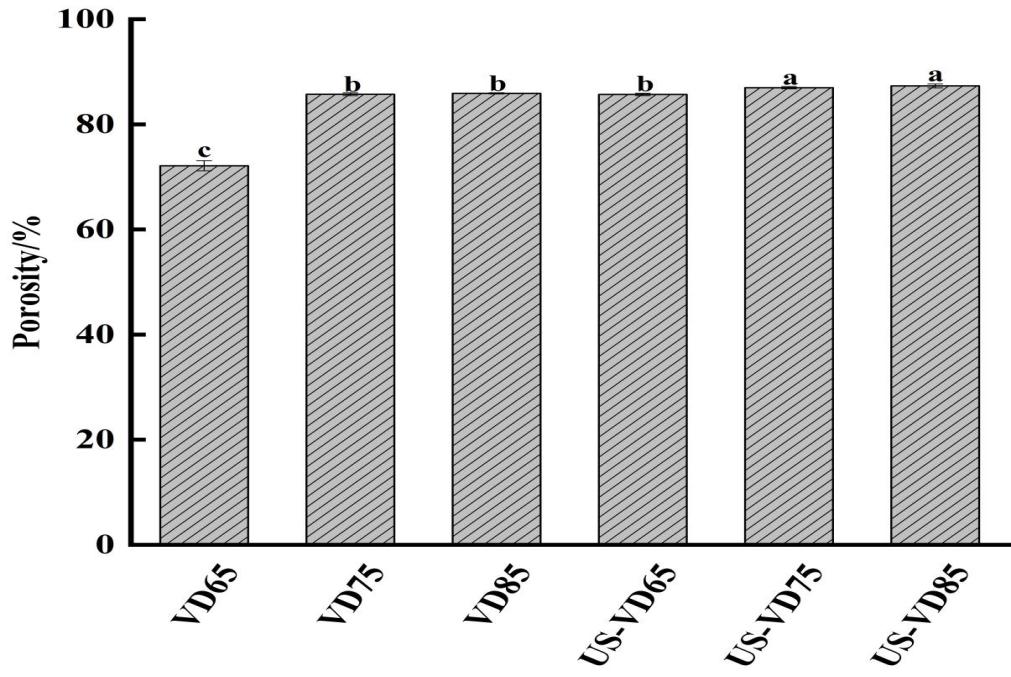


**S-Fig. 2.** Porosity of different dried grapes (VD65, vacuum drying at 65 ℃; VD75, vacuum drying at 75 ℃; VD85, vacuum drying at 85 ℃; US-VD65, vacuum drying at 65℃ after ultrasonic pretreatment; US-VD75, vacuum drying at 75 ℃ after ultrasonic pretreatment; US-VD85, vacuum drying at 85 ℃ after ultrasonic pretreatment).
