# Supplementary figures and images for: Ultrasonic pretreatment and drying temperature-induced modifications of three pectin fractions affect the microstructure and textural properties of dried grapes
Source: Food Chem X. 2025 Jun 4;28:102633. doi: 10.1016/j.fochx.2025.102633 (PMC12173666; doi:10.1016/j.fochx.2025.102633)

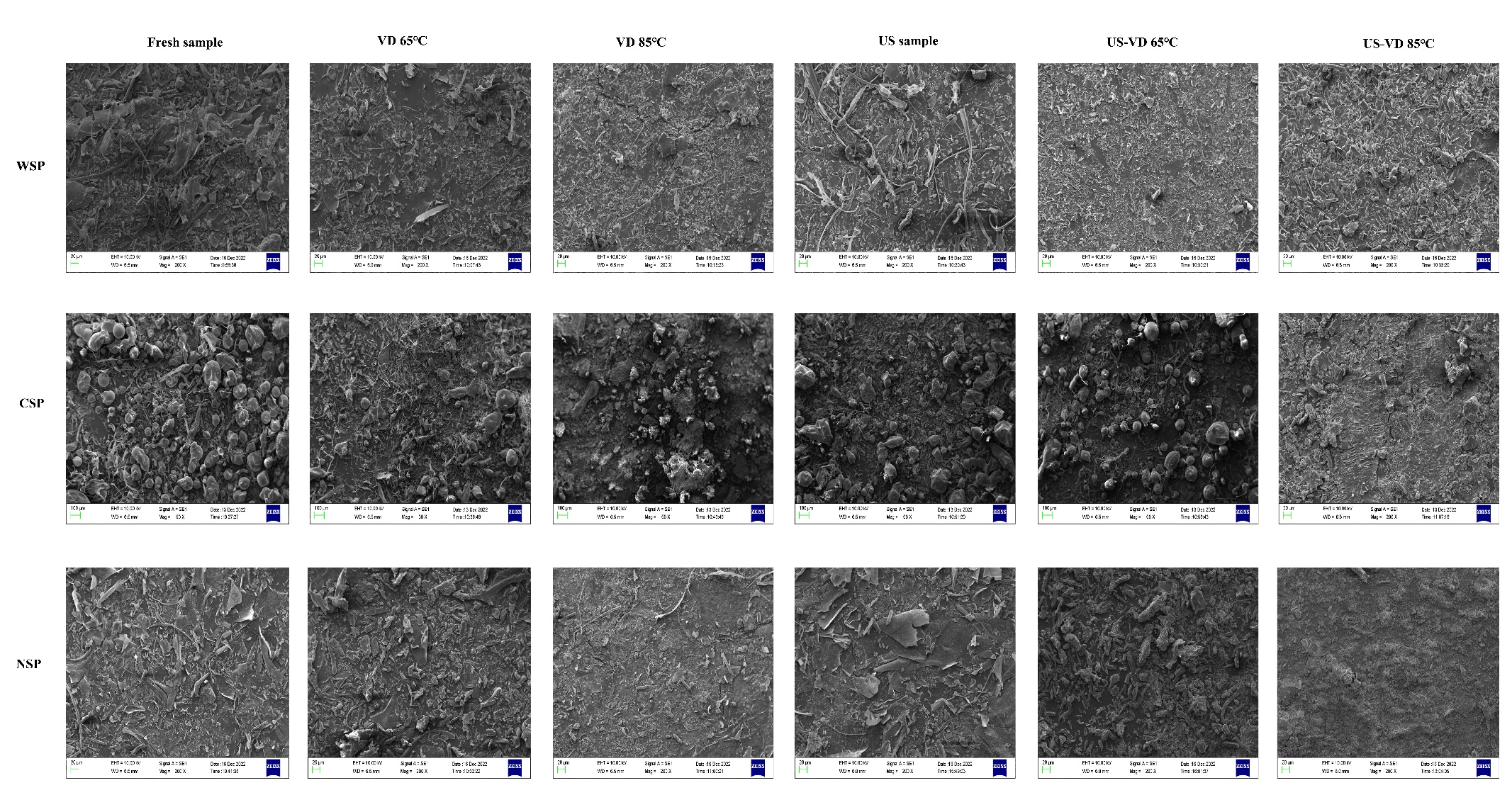


**S-Fig. 3.** Microstructure of three pectin fractions in different dried grapes

Supplement: Supplementary file 3 — Supplementary material 3 [file mmc3.docx]
