# Supplementary material for: Ultrasonic pretreatment and drying temperature-induced modifications of three pectin fractions affect the microstructure and textural properties of dried grapes
Source: Food Chem X. 2025 Jun 4;28:102633. doi: 10.1016/j.fochx.2025.102633 (PMC12173666; doi:10.1016/j.fochx.2025.102633)
Supplement: Supplementary file 4 — Supplementary material 4 [file mmc4.docx]

S-Table 1 The standard curve of neutral sugar

| Sugar name | Equation | R^2^ |
| --- | --- | --- |
| Rha | Y=7014.7X-123.98 | 0.9978 |
| Ara | Y=7712.9X-59.59 | 0.9999 |
| Gal | Y=8420.5X-512.93 | 0.9993 |
| Glu | Y=8384.2X-1052.0 | 0.9987 |
| GluA | Y=7453.4X-104.39 | 0.9994 |
| Man | Y=8289.2X+7.00 | 0.9995 |

Note: Rha-rhamnose, Ara-arabinose, Gal- galactose, Glu- glucose, GluA-glucuronic acid, Man- mannose.
